# Effect of Pathogenic Fungal Infestation on the Berry Quality and Volatile Organic Compounds of Cabernet Sauvignon and Petit Manseng Grapes

**DOI:** 10.3389/fpls.2022.942487

**Published:** 2022-07-22

**Authors:** Xueyao Li, Tinggang Li, Minmin Li, Deyong Chen, Xiaowei Liu, Shanshan Zhao, Xiaofeng Dai, Jieyin Chen, Zhiqiang Kong, Jianxin Tan

**Affiliations:** ^1^College of Food Science and Technology, Hebei Agricultural University, Baoding, China; ^2^State Key Laboratory for Biology of Plant Diseases and Insect Pests, Institute of Plant Protection, Chinese Academy of Agricultural Sciences, Beijing, China; ^3^Shandong Academy of Grape, Shandong Academy of Agricultural Sciences, Jinan, China; ^4^Key Laboratory of Agro-Products Quality and Safety Control in Storage and Transport Process, Ministry of Agriculture and Rural Affairs, Institute of Food Science and Technology, Chinese Academy of Agricultural Sciences, Beijing, China; ^5^College of Life Sciences, Tarim University, Alar, China

**Keywords:** Cabernet Sauvignon, Petit Manseng, GC-IMS, volatile organic compounds, pathogenic fungal infestation

## Abstract

The effect of pathogenic fungal infestation on berry quality and volatile organic compounds (VOCs) of Cabernet Sauvignon (CS) and Petit Manseng (PM) were investigated by using biochemical assays and gas chromatography-ion mobility spectrometry. No significant difference in diseases-affected grapes for 100-berry weight. The content of tannins and vitamin C decreased significantly in disease-affected grapes, mostly in white rot-affected PM, which decreased by 71.67% and 66.29%. The reduced total flavonoid content in diseases-affected grape, among which the least and most were anthracnose-affected PM (1.61%) and white rot-affected CS (44.74%). All diseases-affected CS had much higher titratable acid, a maximum (18.86 g/100 ml) was observed in the gray mold-affected grapes, while only anthracnose-affected grapes with a higher titratable acid level (21.8 g/100 mL) were observed in PM. A total of 61 VOCs were identified, including 14 alcohols, 13 esters, 12 aldehydes, 4 acids, 4 ketones, 1 ether, and 13 unknown compounds, which were discussed from different functional groups, such as C6-VOCs, alcohols, ester acetates, aldehydes, and acids. The VOCs of CS changed more than that of Petit Manseng’s after infection, while gray mold-affected Cabernet Sauvignon had the most change. C6-VOCs, including hexanal and (*E*)-2-hexenal were decreased in all affected grapes. Some unique VOCs may serve as hypothetical biomarkers to help us identify specific varieties of pathogenic fungal infestation.

## Introduction

Grape (*Vitis vinifera L.*) is one of the oldest horticultural plants in the world that existed since prehistoric times and have survived to the present day (Aşçi et al., 2021; Taskesenlioglu et al., 2022). This species, apart from being one of the most extensively cultivated fruit trees in the world due to its rich biochemical content, is also a fascinating subject for history and evolutionary studies (Kupe et al., 2021). The grape has also been the source of not only nutrition but also of beliefs and symbols in people’s daily lives throughout history. Pathogenic fungal infestation is one of the main reasons affecting the development of the grape and wine industry, causing serious economic losses (Alkan et al., 2021). Common pathogenic fungal infestations include gray mold (*Botrytis cinerea*), white rot [*Coniothyrium diplodilla (Speg.) Sacc*], anthracnose (*Colletotrichum sp.*), and others. Gray mold, which is caused by *Botrytis cinerea*, usually kills and destroys berries, resulting in serious losses to many crops (Agudelo-Romero et al., 2015; Rastgou et al., 2022). Grape anthracnose, commonly known as grape bitter rot, causes the fruit to rot and the leaves to develop leaf spots because of infection. White rot often induces serious harm to fruit, which occurs in high temperature and high humidity environments. These pathogenic fungal infestations seriously affect the yield and quality of grapes, resulting in potential harm to the human body and commercial losses in the grape industry. However, precise interactions between various pathogenic fungal infestations and grapes have not been fully explored. Investigating various pathogenic fungal infestations of grapes during growth is critical for exploring the effects of the pathogenic fungal infestations and offering further information on interactions between pathogenic fungi and grapes.

As two popular wine grape varieties in the world, Cabernet Sauvignon (CS) and Petit Manseng (PM) have the exquisite aroma and noble quality as raw materials for red wine and white wine. At present, the serious problem is that pathogenic fungal infestations have negative impacts on the berry’s quality and composition. Pathogenic fungal infestations usually lead to rapid physiological changes in berries, such as weight loss, skin color fading, tissue softening, and shortening of shelf-life, which badly reduces the market value of grapes (Vazquez-Hernandez et al., 2018). In addition to such visible quality characteristics, the nutritional value and chemical constituents of grapes will be changed, including more microbial metabolites, sugar degradation, and acid production (Solairaj et al., 2021). Santos et al. (2022) found that Trincadeira wine grapes showed serious symptoms after *Botrytis cinerea* infection, the content of total phenol and total anthocyanin was greatly lower than in healthy samples. An infection experiment conducted by Pons et al. (2018) confirmed that, for Merlot wines, pH and total acidity were the parameters that were systematically influenced by *P. viticola* infection. However, changes in fruit quality are sometimes associated with defensive behavior. Braga et al. (2019) found that phenolic compounds accumulate in infected areas compared with healthy areas, thus, indicating the accumulation of total phenols in resistance response.

Wine aroma is a key criterion for assessing the quality of wine, and the sources of aroma substances in wine are diverse. Among them, variety aroma is the aroma that comes directly from the grapes and characterizes the wine’s typicality and origin style. Wine aroma is primarily influenced by the aroma of the varieties, such as volatile organic compounds (VOCs), which are produced by grape metabolism (Dudareva et al., 2013). VOCs include organic categories, such as alcohols, aldehydes, esters, fatty acids, and benzenes, which contribute to producing subtle aroma differences. Simultaneously, pathogenic fungal infestations change the aroma components of grapes by altering VOCs. Pinar et al. (2017) reported that bunch rot mainly caused an increase in the intensities of peach-like/fruity, floral, and liquor-like/toasty aroma notes, which were shown to be related to variations in aroma composition, mainly a modest increase of esters and alcohols. A previous study (Guerche et al., 2006) showed that *Botrytis cinerea* could promote to metabolize some trace volatile substances to produce odor in wine, the volatile metabolites detected in infected grape were mainly 2-methylisoborneol, 1-octene-3-ol, 1-octene-3-one, 2-octene-1-ol, and 2-heptanol. Also, Gadoury et al. (2007) found that powdery mildew accelerated the formation of other pathogenic fungal infestations and increased the contents of ethyl acetate, acetic acid, and ethanol in wine. On the other hand, some experimental trials have demonstrated the capacity of various VOCs produced by plants to inhibit germination and growth of plant pathogens. It has been reported that *Botrytis cinerea* was highly sensitive to the *in vitro* application of monoterpenes, such as (+)-limonene (Simas et al., 2017). However, exposure to (+)-limonene stimulated *in vitro* growth of *Penicillium digitatum*, whereas this fungus was highly inhibited by the application of citral (Simas et al., 2017). Lazazzara et al. (2018) demonstrated that *P. viticola* infection was inhibited in leaf tissues by some VOCs, such as 2-ethylfuran, 2-phenyl ethanol, β-cyclocitral, or trans-2-pentena. In addition, the growth of *Colletotrichum acutatum*, causing citrus post-bloom fruit drop, was moderately inhibited *in vitro* when exposed to linalool (Marques et al., 2015). Consequently, the elucidation of the changes in VOCs of grapes suffering pathogenic fungal infestations during growth is a task of highly practical significance to further explore the impact of pathogenic fungal infestations on grapes.

Gas chromatography-ion mobility spectrometry (GC-IMS) is an advanced technology for the analysis of VOCs, which combines the high separation ability of GC with the rapid response characteristics of IMS (Tuzimski et al., 2016). GC-IMS has the advantages of fast detection speed, simple operation, and easy sample preparation steps (Rousserie et al., 2020). GC-IMS shows rapid response and high sensitivity in the detection of trace volatile and semi-volatile organic compounds in different matrices. Zhao et al. (2022) used GC–IMS with gas chromatography-mass spectrometry technology to identify the flavor components qualitatively and quantitatively, under the various treatment of chiral tebuconazole on the flavor of Merlot and Cabernet Sauvignon wines. GC-IMS shows good application value in flavor analysis, and it is a new technology to detect the changes in volatile components during food processing and storage.

This study aims to investigate the impacts of the various pathogenic fungal infestations on two wine grapes during their growth. To be more specific, biochemical assays and GC-IMS were used to further explore the effects of pathogenic fungal infestations on berry quality and VOCs in Cabernet Sauvignon and Petit Manseng, then evaluate the influences of gray mold, white rot, and anthracnose. In addition, the VOCs were analyzed from three aspects: (1) pathogenic fungal infestations, (2) volatile compound types, and (3) wine grape varieties. The obtained results provide comprehensive and reliable information for assessing the impacts of pathogenic fungal infestations in wine grapes.

## Materials and Reagents

### Chemicals and Reagents

The gallic acid, hesperidin, and L (+)-ascorbic acid analytical standards with purities of > 99% were provided by Dr. Ehrenstorfer GmbH (LGC Standards, Augsburg, Germany). Acetone, sodium carbonate, Folin-phenol, diethylene glycol, citric acid, glacial acetic acid, boric acid, metaphosphoric acid, sodium acetate trihydrate, o-Phenylenediamine, thymol blue, activated carbon, sodium tungstate dihydrate, sodium molybdate dihydrate, lithium sulfate, NaOH, HCl, H_3_PO_4_, and H_2_SO_4_ were purchased from Sinopharm Chemical Reagent Co., Ltd. (Beijing, China); all chemicals were analytical grade (> 99%) unless otherwise stated. All standard solutions were stored in brown glass bottles wrapped in aluminum foil to avoid light exposure. Before analysis, the bottles were stored at 4°C. Under these conditions, no degradation was observed for 3 months.

### Plant Materials and Treatments

The Cabernet Sauvignon and Petit Manseng grapes used in this study were cultivated in Yantai, Shandong Province, China (E121.39, N37.52). The grapevine trees of Cabernet Sauvignon and Petit Manseng were both 6 years old, with row spacing of 1.8 m × 0.5 m and 1 m × 2 m, respectively. Naturally infected berries with similar severity collected from the vineyard were used to identify consistent berry responses to pathogenic fungal infestations across natural conditions. Grapes were taken after veraison when the berry started to show fungal infection symptoms, and each selected bunch was submitted to a pathological examination for identifying the fungal infection before sample collection. After this, the infection degree of the grapes we chose was as follows: corresponding to healthy tissues and clusters of small lesions (diameter < 2 mm), there were hyphae in the early stage of development and hyphae structure in the middle stage (rarely visible and carefully observed). For CS and PM, the treatment group consisted of grapes with three pathogenic fungal infestations: gray mold (CS-GM, PM-GM), anthracnose (CS-AN, PM-AN), and white rot (CS-WR, PM-WR). The healthy samples were selected as the control groups (CS-CK, PM-CK). Grapes were wrapped in wet gauze after harvest and brought back to the laboratory immediately after refrigeration in a preservative box. The samples were placed in a 4°C-refrigerator before measurement and crushing. All measurements include three replicates, each containing three random clusters. For each grape cluster, the grapes were randomly selected from the shoulder, middle, and bottom of the cluster. One hundred-berry weight, particle size, soluble solids content, titratable acid, and the content of total phenolics, total flavonoids, vitamin C, and tannins were determined.

### Determination of Physicochemical Parameters

#### One Hundred-Berry Weight and Particle Size

One hundred grape berries in each group were chosen randomly to measure 100-berry weight (g), then washed with distilled water and dried by the filter. It was measured by an electronic balance; measurements were repeated three times. Vernier calipers were used to measure the particle size of ten grape berries in each group.

#### Soluble Solid Content and Titratable Acid

The clear juice (supernatant) extracted was used to determine SSC by using a manual refractometer (ATAGO Company, Fukuoka, Japan) and the results were recorded as the degree of°Brix. TA was determined by titration using 10 ml of diluted juice with the addition of NaOH (0.1 N) and two drops of phenolphthalein until a light pink color was formed (30 s without fading). Finally, the numerical value was expressed by the predominant acid.

### Determination of the Phenolic Compounds and Vitamin C in Grapes

#### Total Phenolics

Total phenolics (TP) content was determined by the modified Folin method (Thimmaiah, 1999). The gallic acid standard sample was dissolved in distilled water and diluted to obtain a standard solution of 0.05 mg/ml. Then, 1 mL (1.2, 1.4, 1.6, 1.8, and 2) of the standard solution was accurately measured, and 0.5 mL of Folin-phenol reagent and 0.5 mL of 10% sodium carbonate solution were added. The distilled water was diluted to 10 ml, and the solution was reacted in a water bath at 25°C for 1 h. The absorbance was measured at 765 nm with the blank reagent as the control to establish the standard curve.

Four-gram sample was weighed, 16 ml of 70% acetone was added, and then, the supernatant was extracted for 3 h and centrifuged for 10 min at 10,000 rpm. A total of 0.2 ml of the sample solution was taken, added with 0.5 ml of Folin-phenol reagent. Then,0.5 ml of 10% sodium carbonate solution was added and was reacted in a water bath at 25°C for 2 h. The absorbance was measured at 765 nm to obtain the TP content.

#### Total Flavonoids

The experiment was designed to determine Total flavonoids (TF) content following the guideline (NY/T, 2010-2011). The standard solutions of 0 ml, 1 ml, 2 ml, 3 ml, 4 ml, and 5 ml of hesperidin (200 mg/L) were absorbed into the test tubes, and then, 5 ml of 90% diethylene glycol solution and 0.1 ml of NaOH (4 M) solution were added. The standard solution (0 mg/L, 20 mg/L, 40 mg/L, 60 mg/L, 80 mg/L, and 100 mg/L) was incubated in a water bath at 40°C for 10 min, and then cooled for 5 min. The absorbance was measured at 420 nm by ultraviolet spectrophotometer (Shimadzu UV-2450, Kyoto, Japan), and the standard curve was drawn. Five grams of sample was mixed with NaOH solution, and the PH was adjusted to 13.0. The PH was adjusted by the citric acid solution (20% w/v) to 6 after 30 min, and 5 ml of the solution was mixed with 5 ml of diethylene glycol solution and 0.1 ml of NaOH solution. The absorbance value was determined using the standard curve to calculate the mass concentration of TF.

Total flavonoids (TF) was calculated by hesperidin mass fraction ω, and the value was expressed as mg/100 g using the following formula:


(1) $$\omega =\frac{\rho \times 10\times 100\times 1000}{m\times V\times 100}$$

where ρ is hesperidin mass concentration (mg/L); *V* is determination of absorbed test liquid volume (mL); *m* is sample weighing mass (g); 10 is color constant volume (ml); 100 is sample extraction volume (ml).

#### Vitamin C

The VC content of grapes was measured by using the fluorescence method based on (GB 5009.86, 2016). One hundred grams of the grape extract was homogenized after adding 100 g of metaphosphate-acetic acid solution, diluted with the metaphosphate-acetic acid solution, or metaphosphate-acetic acid-sulfuric acid solution. The pH was adjusted to 1.2, 50 ml of supernatant was mixed with 2 g of activated carbon after filtering, and two groups of 10-ml filtrates were taken and added with 5 ml of sodium acetate solution (50% w/v) and 5 ml of boric acid-sodium acetate solution as “sample solution and “sample blank solution,” respectively. The ascorbic acid’s standard working solution was treated in the same way as “standard solution” and “standard blank solution.”

First, 0.5 ml, 1 ml, 1.5 m, and 2 ml of standard solution were absorbed and supplemented with water to 2 ml. In addition, 2 ml of “standard blank solution” was mixed with 5 ml of phthalediamine solution in the darkroom, and the reaction was carried out at room temperature for 35 min. Finally, the fluorescence intensity was measured at 338 nm and 420 nm, and the standard curve was drawn. Two milliliters of “sample solution” and “sample blank solution” with 5 ml of phthalediamine solution were reacted in the darkroom for 35 min reaction at room temperature. The result was measured and the total amount of L (+)-ascorbic acid was determined according to the standard curve. The results were expressed as mg/100 g using the following formula:


(2) $$X=\frac{c\times V}{m}\times F\times \frac{100}{1000}$$

where *X* is total L (+)-ascorbic acid in sample (mg/100 g); *c* is L (+)-ascorbic acid mass concentration (μg/mL); *V* is sample volume (mL); *m* is actual sample quality (g); *F* is the sample solution dilution ratio; 100 is conversion coefficient; 1,000 is conversion coefficient.

#### Tannins

The tannins content of grapes was measured by using a spectrophotometric method based on (NY/T, 1600-2008). Sodium tungstate-sodium molybdate mixed solution was configured, then 1 ml of 0.00 mg/L, 10 mg/L, 20 mg/L, 30 mg/L, 40 mg/L, and 50 mg/L gallic acid standard solution was absorbed, 5 ml water was added, 1 ml of sodium tungstate-sodium molybdate mixed solution, and 3 ml of sodium carbonate solution (7.5% w/v) were mixed with 0 mg/L, 1 mg/L, 2 mg/L, 3 mg/L, 4 mg/L, and 5 mg/L standard solution. After coloration, the absorbance at 765 nm was measured and the standard curve was plotted. Five grams of sample centrifuged at 8,000 rpm for 4 min after hot water bath extraction, 1 ml of extract was taken and added with 5 ml of water, 1 ml of sodium tungstate-sodium molybdate mixed solution, and 3 ml of sodium carbonate solution. The absorbance at 765 nm was measured, and the concentration of tannin was calculated according to the standard curve. The tannin content in the sample (calculated by gallic acid) was calculated as follows:


(3) $$\omega =\frac{\rho \times 10\times 10\times A}{m}$$

where ω is tannin content in samples (mg/100 g); ρ is gallic acid concentration in the determination solution (mg/L); 10 is the constant volume (ml); 10 is the conversion coefficient; *A* is the sample dilution multiples; m is the sample quality (g).

### Volatile Analysis by Gas Chromatography-Ion Mobility Spectrometry

For volatile analysis, the grape sample was homogenized, and 5 g was accurately weighed, then placed in a 20-ml headspace vial and incubated at 40°C for 20 min before sampling. The analysis was performed by using headspace-gas chromatography-ion mobility spectrometry (HS-GC-IMS) (FlavourSpec^®^, G.A.S., Dortmund, Germany). The grape samples were incubated at 500 r/min for 20 min at 40°C. Thereafter, 500 μL of gas from the headspace was automatically infused into the heated injector by a syringe in a splitless mode at 85°C. At that point, the samples were directed into an MXT-WAX capillary column (30 m × 0.53 mm × 1 μm, Restek Corporation, Bellefonte, PA, United States). Purified nitrogen (99.999% purity) with a flow rate of 150 ml/min was used as the drift gas for IMS. The temperature of the column and the IMS was 60°C and 45°C, respectively. The carrier gas followed a programmed flow: 2 ml/min for 2 min, increased to 100 ml/min within 20 min, then kept at 100 ml/min for 10 min.

The eluted analytes ionization source was 3H ionization, driven to a drift tube which was run at a constant temperature of 45°C and voltage of 5 kV. C4-C9 *n*-ketones (Sinopharm Chemical Reagent Beijing Co., Ltd., China) was used as references when the retention index (RI) was calculated. Volatile compounds were analyzed according to the differences in their RI and drift time (DT) in the GC × IMS Library from different perspectives.

### Date Analysis

A significant difference was evaluated by one-way analysis of variance (ANOVA) followed using Duncan’s multiple range test with a significant level (*P* < 0.05). IBM SPSS statistics (version 20.0, SPSS Inc., Chicago, IL, United States) was employed for significance analysis. The scattered boxplot and principal component analysis (PCA) were implemented by Origin 2021 from Origin Laboratories (available at www.originlab.com). Heatmap with clustering analysis was made by TBtools software (Chen et al., 2020).

The instrument analysis software was composed of Laboratory Analytical Viewer (version 2.2.1, G.A.S. Dortmund, Germany) with its plug-ins: Reporter and Gallery Plot, and GC × IMS Library Search, which were applied to qualitative and comparative detection.

## Results and Discussion

### Effects of Pathogenic Fungal Infestations on Physicochemical Parameters of Cabernet Sauvignon and Petit Manseng

#### One Hundred-Berry Weight and Particle Size

**FIGURE 1:**
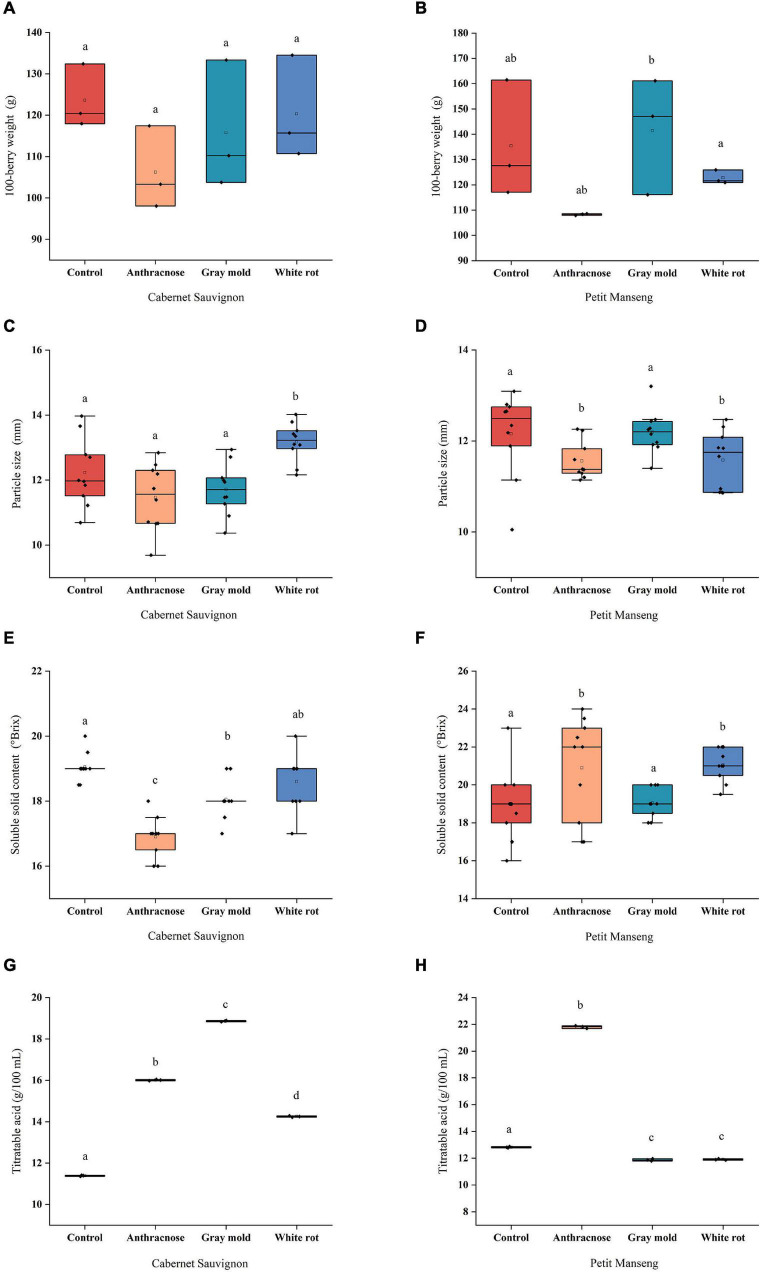
The scattered boxplots show physicochemical parameters of Cabernet Sauvignon and Petit Manseng grapes: **(A, B)** 100-berry weight; **(C, D)** Particle size; **(E, F)** Soluble Solid Content (SSC); **(G, H)** Titratable acid (TA). Different letters (lower case) on the top of the bars represent significant differences among the investigated grape samples. Scattered boxplots show individual data points, median, average value, 25th, and 75th percentile.

Berry size is widely considered to be a factor in determining the quality of wine grapes. As shown in Figures 1A–D. In our study, there were no significant differences between the disease-infected grapes and healthy grapes for the 100-berry weight of CS and PM, while most infected groups decreased slightly. The results showed that the grape infected with anthracnose decreased slightly compared with other disease groups, CS-AN and PM-AN were decreased by 14.03% and 20.02%, respectively. On the other hand, a slight increase was observed in 100-berry weight and particle size for PM, which were 4.49% and 0.34%, respectively, after infection with *Botrytis cinerea*. The effect of pathogenic fungal infestation on berry weight depends on the degree of ontogenic resistance expressed by berries when infected by pathogenic fungi. Gadoury et al. (2003) showed that once the berries reached the diameter of 3 mm (28 days after flowering), powdery mildew did not significantly reduce the weight of the berries. However, the weight of berries was significantly reduced and colonized by the pathogen heavily when berries were infected before resistance. It is speculated that the unobvious change of berry weight may be related to the resistance of grapes when pathogenic fungal infections occurred. The comparison of 100-berry weight and particle size showed that CS-WR > CS-GM > CS-AN and PM-GM > PM-WR > PM-AN.

#### Soluble Solid Content and Titratable Acid

Soluble solid content (SSC) and TA are related to the taste of wine, which served as important indexes to reflect the quality of the berry and the disease resistance. Figures 1E–H show the SSC and TA of the two wine grapes infected with various pathogenic fungi. In CS, the SSC of the infected samples was lower than CS-CK, and CS-AN and CS-GM significantly decreased (*P* < 0.05) by 11.29% and 5.25%, respectively, compared with the healthy samples. Meanwhile, different pathogenic fungal infestations cause a rise of TA content in CS to variable degrees: gray mold (18.86 g/100 ml)>anthracnose (16.01 g/100 ml) > white rot (14.25 g/100 ml). However, the SSC increased after infection in PM, and the maximum (21.80 g/100 ml) of the TA was observed in the PM-AN, and other disease groups were lower than in the healthy group. In a previous study conducted by Stummer et al. (2003), for Cabernet Sauvignon infected with powdery mildew in 2001, the SSC with infection degree greater than 30% has decreased significantly, which was speculated to be related to the high level of powdery mildew infection hindering sugar accumulation, and the TA increased or decreased under different infection degrees. On the other hand, the explanation for the increase in sugar concentration after infection may be associated with the decrease in the volume of diseased berries and the increase in transpiration water loss (Calonnec et al., 2004). Moreover, Girardello et al. (2020) showed that the acidity of Chardonnay grapes increased after infection with erythema, which was supposed to be related to the high concentration of potassium (K) in juice. In summary, it was inferred that our results may be caused by many reasons such as the fruit year, grape variety, pathogenic fungal infestation, the degree of the infection, and so on.

### Effects of Pathogenic Fungal Infestation on the Content of the Phenolic Compounds and Vitamin C in Cabernet Sauvignon and Petit Manseng

#### Total Phenolics

**FIGURE 2:**
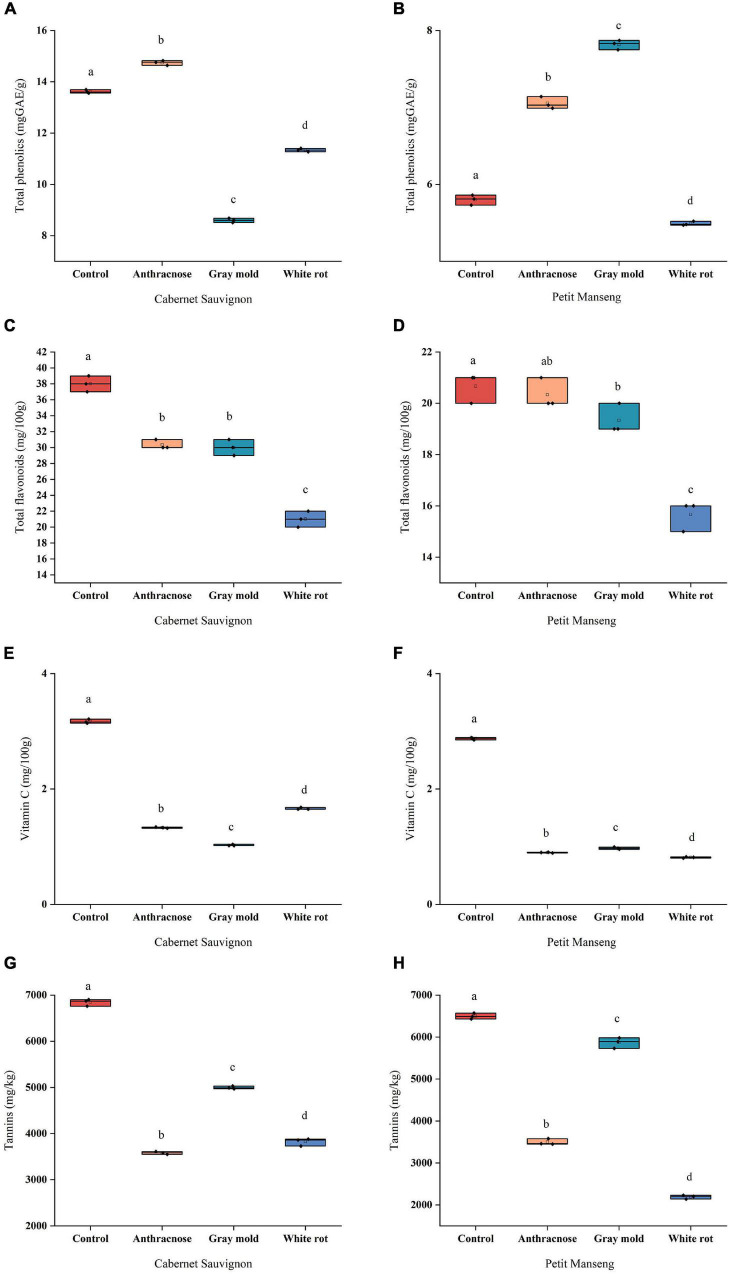
The scattered boxplots show the content of the phenolic compounds and vitamin C in all samples of Cabernet Sauvignon and Petit Manseng grapes: **(A, B)** Total phenolics (TP); **(C, D)** Total flavonoids (TF); **(E, F)** Vitamin C; **(G, H)** Tannins. Different letters (lower case) on the top of the bars represent significant differences among the investigated grape samples. Scattered boxplots show individual data points, median, average value, 25th, and 75th percentile.

Grape is rich in phenols, and plant polyphenols have been proven to have potential antibiosis activity, which is mainly distributed in the skin, stems, leaves, and seeds of the grape, rather than the juicy middle part (Bruno and Sparapano, 2007). The phenolic compounds mainly include proanthocyanidins, anthocyanins, flavonols, resveratrol, and phenolic acids. As shown in Figures 2A,B, for healthy grapes, the TP of CS (13.62 mg GAE/g) was much higher than PM (5.80 mg GAE/g). In CS and PM, a significant difference (*P* < 0.05) was observed in all disease groups compared to the healthy grapes. In comparison to CK, the TP content of the grapes infected with anthracnose increased by 8.25% and 21.61% significantly, while it decreased after infection with white rot. It is worth noting that the TP content in CS and PM presented the opposite effects after infection with gray mold, which showed that CS decreased by 5.02 mg GAE/g but PM increased by 2.02 mg GAE/g compared with CK. A study reported that total phenolic extracted in methanol had a visible downward tendency after infection, and total phenolic extracted in water, mainly hydrophilic compounds such as hydroxybenzoic acid, anthocyanin, flavonoids, or tannin, decreased after infection (Santos et al., 2022). However, wines made from powdery mildew-affected grapes generally have higher phenolic levels than wines made from unaffected grapes (Steel et al., 2013). Bruno and Sparapano (2007) reported that the content of total phenol in grape skins varies with species, soil composition, climate, geographical origin, cultivation methods, and infection exposure. Therefore, it is speculated that different changes were impacted by a range of factors.

#### Total Flavonoids

Flavonoids have strong antioxidant properties and could be measured to reflect the antioxidant capacity of plants (Figures 2C,D). The CS-CK (38 mg/100 g) and PM-CK (20.67 mg/100 g) both had the highest content of TF, and almost infected grapes were significantly decreased (*P* < 0.05), except for PM-AN. The TF content decreased to a similar extent after infection with anthracnose and gray mold, the results showed that the content of CS-AN and CS-GM was 30.33 mg/100 g, 30 mg/100 g, and PM-AN and PM-GM were 20.33 mg/100 g, 19.33 mg/100 g, respectively, which suggested that anthracnose and gray mold might have similar effects on the TF. Moreover, flavanols are the most ubiquitous flavonoids in foods, previous study focused on the determination of flavonols of Zinfandel grapes, the findings proved that infected grapes had a lower flavonol content compared to the control group (Blanco-Ulate et al., 2017).

#### Vitamin C

Vitamin C (VC) is the primary aqueous antioxidant that effectively reduces the damage that reactive oxygen species (ROS) produced. The maximum was found in the control group both in the PM and CS (Figures 2E,F). After being invaded by pathogenic fungi, the content of VC in CS and PM decreased significantly (*P* < 0.05), which indicated that the invasion of pathogenic fungi caused a great deal of degradation of VC in grapes, which damaged the nutritional quality. Murria et al. (2018) found that the contents of chlorophyll, carotenoids, and ascorbic acid in infected leaves are lower than those in healthy leaves.

#### Tannins

Tannins are one of the key factors determining the quality of grapes and wines, which can decide the sensory attributes of wines, such as color, taste, astringency, and bitterness by the content and proportion (Rousserie et al., 2020). As shown in Figures 2G,H, There was a significant difference (*P* < 0.05) in tannins of infected grapes compared to healthy grapes in the CS and PM, pathogenic fungal infestation reduced the tannins to a great extent. The grape berry analyses performed in 2010 showed that it greatly decreases skin procyanidins concentrations after infection; it should be noted that the total tannin content in pericarp tissue is considered to contribute to the pre-resistance of berries to pathogenic fungal infestation (Deytieux-Belleau et al., 2009). Previous reports speculated that the tannins were closely related to climatic factors, resulting in stronger responses of plants and more tannins in a warmer environment (Cauduro Girardello et al., 2020). Moreover, tannin has also a certain correlation with grape maturity (Rousserie et al., 2020).

### Volatile Organic Compounds Analysis by Gas Chromatography-Ion Mobility Spectrometry

Volatile organic compounds (VOCs) play an indispensable role in the key metabolic pathways involved in plant growth, development, reproduction, and defense (Bouwmeester et al., 2019). Once the fruit was infected by pathogenic fungal, VOCs can be used as toxins, defense compounds, energy sources, and infection enhancers (Santos et al., 2022). Understanding the alterations of fruit to pathogenic fungal infestation is essential for the improvement of grapes and for the sustainability of wine production. In this study, VOCs were analyzed from three different aspects: pathogenic fungal infestation, VOC types, and wine grape varieties.

#### Effects of Pathogenic Fungal Infestations on Volatile Organic Compounds

**FIGURE 3:**
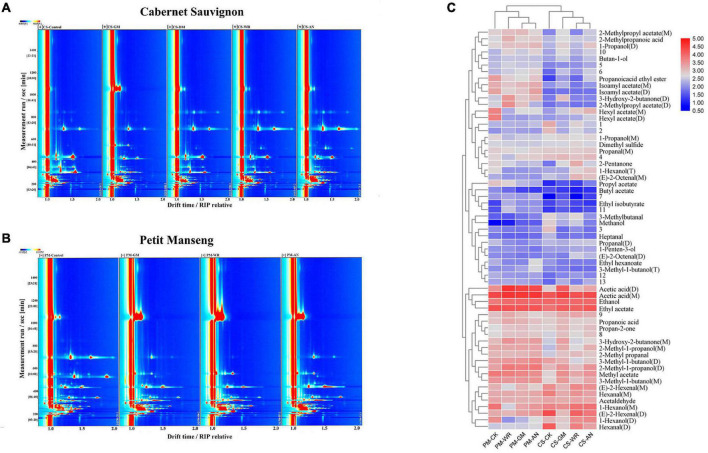
**(A, B)** Comparison of GC-IMS spectra and **(C)** Heatmap with clustering analysis of VOCs. The numbers in panel **(A, B)** represent VOCs with large differences (the compound names are shown in Supplementary Table 1). **(C)** shows the changes in VOCs induced by pathogenic fungal infestation with the control group in Cabernet Sauvignon and Petit Manseng wine grapes.

The GC-IMS spectra of the VOCs in the samples are shown in Figures 3A,B, each spectrum represents the sample for each treatment, the Y-axis represents the retention time (s) of gas chromatography, and the X-axis represents the ion migration time. The red vertical line at X-axis 1 is the reactive ion peak (RIP), on both sides of this peak, each point represents a volatile organic compound. The concentration of VOCs was determined by color, blue was the background color, white represents low concentration, and red represents high concentration, that is, the deeper the color, the greater the concentration. It can be intuitively found from the spectrum that after pathogenic fungal infestations, the VOCs of CS and PM both had significant changes. A total of 61 volatile compounds, composed of 14 alcohols, 1 ether, 12 aldehydes, 4 acids, 4 ketones, 13 esters, and 13 unknown compounds, were simultaneously identified (including monomers and polymers) in the samples of CS and PM (Table 1 and Supplementary Table 1), and most of the volatile substances were alcohols, esters, and aldehydes. Among all infected grapes, the variation of VOCs in CS-GM has the greatest difference compared with others. In this group, the concentrations of 9 VOCs decreased, including 6 aldehydes, 2 alcohols, and 1 ether. The concentrations of 19 VOCs increased, including 8 esters, 3 ketones, 3 acids, 4 alcohols, and 1 aldehyde. Among them, 2-methylpropanoic acid, ethyl isobutyrate, 3-hydroxy-2-butanone, acetic acid, and propionic acid increased significantly. As for PM-GM, the concentration of 14 VOCs reduced, including 8 esters, 2 alcohols, 3 aldehydes, and 1 ether, and 11 VOCs were raised, including 4 alcohols, 3 acids, 2 esters, and 2 ketones. Notably, previous studies had explanations for the reduction in 1-hexanol, it is reported that this may be due to the production of noble root wines, which is the result of a unique physiological process (Tosi and Azzolini, 2013). Noble root wine is a sweet wine formed by *Botrytis cinerea* infecting grapes under specific growth conditions. Therefore, we speculated that the grapes infected with *Botrytis cinerea* would generate noble root wine due to certain development circumstances.

**TABLE 1:** Compositions of the volatile substances determined by gas chromatography-ion mobility spectrometry (GC-IMS) analysis.

Count	Compound	CAS#	Formula	MW	RI*^a^*	Rt*^b^*	Dt*^c^*
1	2-Methylpropanoic acid	C79312	C_4_H_8_O_2_	88.1	1566.6	1717.688	1.15764
2	Propanoic acid	C79094	C_3_H_6_O_2_	74.1	1538.4	1499.028	1.10708
3	acetic acid	C64197	C_2_H_4_O_2_	60.1	1462.8	1049.26	1.05129
4	acetic acid*	C64197	C_2_H_4_O_2_	60.1	1464.7	1058.641	1.15766
5	1-Hexanol	C111273	C_6_H_14_O	102.2	1368.4	701.683	1.32825
6	1-Hexanol*	C111273	C_6_H_14_O	102.2	1369.6	704.455	1.65395
7	1-Hexanol**	C111273	C_6_H_14_O	102.2	1368.9	702.792	1.99559
8	3-hydroxy-2-butanone	C513860	C_4_H_8_O_2_	88.1	1298.6	557.923	1.07285
9	3-hydroxy-2-butanone*	C513860	C_4_H_8_O_2_	88.1	1297.0	555.075	1.33145
10	hexyl acetate	C142927	C_8_H_16_O_2_	144.2	1280.5	528.631	1.3847
11	hexyl acetate*	C142927	C_8_H_16_O_2_	144.2	1281.0	529.388	1.90012
12	Ethyl hexanoate	C123660	C_8_H_16_O_2_	144.2	1238.9	468.485	1.3433
13	(*E*)-2-hexenal	C6728263	C_6_H_10_O	98.1	1226.5	451.975	1.1769
14	(*E*)-2-hexenal*	C6728263	C_6_H_10_O	98.1	1227.1	452.709	1.52571
15	1-Penten-3-ol	C616251	C_5_H_10_O	86.1	1168.0	382.14	0.94552
16	isoamyl acetate	C123922	C_7_H_14_O_2_	130.2	1131.6	344.798	1.30098
17	isoamyl acetate*	C123922	C_7_H_14_O_2_	130.2	1131.3	344.509	1.74028
18	2-methyl-1-propanol	C78831	C_4_H_10_O	74.1	1104.4	319.339	1.17187
19	2-methyl-1-propanol*	C78831	C_4_H_10_O	74.1	1103.4	318.471	1.36777
20	Hexanal	C66251	C_6_H_12_O	100.2	1101.5	316.735	1.2624
21	Hexanal*	C66251	C_6_H_12_O	100.2	1094.7	311.238	1.56219
22	2-methylpropyl acetate	C110190	C_6_H_12_O_2_	116.2	1023.4	268.504	1.28717
23	2-methylpropyl acetate*	C110190	C_6_H_12_O_2_	116.2	1021.6	267.449	1.61112
24	2-pentanone	C107879	C_5_H_10_O	86.1	992.8	252.367	1.36789
25	ethanol	C64175	C_2_H_6_O	46.1	935.2	230.801	1.13103
26	propyl acetate	C109604	C_5_H_10_O_2_	102.1	985.8	249.652	1.47729
27	Propanoic acid ethyl ester	C105373	C_5_H_10_O_2_	102.1	964.8	241.659	1.45393
28	Ethyl isobutyrate	C97621	C_6_H_12_O_2_	116.2	973.6	244.977	1.55908
29	ethyl acetate	C141786	C_4_H_8_O_2_	88.1	888.0	214.512	1.32965
30	3-Methylbutanal	C590863	C_5_H_10_O	86.1	922.4	226.276	1.40082
31	Methyl acetate	C79209	C_3_H_6_O_2_	74.1	850.8	202.498	1.19096
32	Propan-2-one	C67641	C_3_H_6_O	58.1	835.8	197.824	1.11461
33	2-Methyl propanal	C78842	C_4_H_8_O	72.1	831.2	196.433	1.28088
34	Propanal	C123386	C_3_H_6_O	58.1	819.6	192.921	1.06504
35	Propanal*	C123386	C_3_H_6_O	58.1	822.0	193.65	1.14125
36	methanol	C67561	CH_4_O	32.0	912.2	222.721	0.98563
37	Acetaldehyde	C75070	C_2_H_4_O	44.1	778.2	180.93	0.98633
38	butyl acetate	C123864	C_6_H_12_O_2_	116.2	1081.9	303.09	1.23772
39	3-Methyl-1-butanol	C123513	C_5_H_12_O	88.1	1213.8	435.646	1.24493
40	3-Methyl-1-butanol*	C123513	C_5_H_12_O	88.1	1214.1	435.999	1.49095
41	3-Methyl-1-butanol**	C123513	C_5_H_12_O	88.1	1215.8	438.118	1.78823
42	1-propanol	C71238	C_3_H_8_O	60.1	1047.9	282.482	1.11188
43	1-propanol*	C71238	C_3_H_8_O	60.1	1048.5	282.816	1.25422
44	(*E*)-2-octenal	C2548870	C_8_H_14_O	126.2	1418.6	852.241	1.34901
45	(*E*)-2-octenal*	C2548870	C_8_H_14_O	126.2	1418.5	851.636	1.81337
46	heptanal	C111717	C_7_H_14_O	114.2	1191.7	408.576	1.34677
47	butan-1-ol	C71363	C_4_H_10_O	74.1	1152.9	366.206	1.18015
48	dimethyl sulfide	C75183	C_2_H_6_S	62.1	799.5	187.004	0.95792

*Dt, drift time; MW, Molecular mass; RI, retention index; Rt, Retention time.*

*^a^Retention index.*

*^b^Retention time.*

*^c^Drift time.*

“*” Represents dimer.

“**” Represents trimer.

In CS-WR, 17 VOCs had a higher level, and 5 VOCs had a lower level compared with healthy grapes, the changes in substances were similar to CS-AN. At the same time, the changes of VOCs in PM-WR and PM-AN were also very close, which speculated that there are some common points in understanding the effects of white rot and anthracnose on grapes. Meanwhile, the contents of acetic acid, 3-hydroxy-2-butanone, propan-2-one, 2-methyl-1-propanol, 1-penten-3-ol, and 1-propanol increased in the CS-WR and PM-WR, which inferred that the changes in aroma components were closely related to the pathogenic fungal infestation. Most esters, dimethyl sulfide, propanal, and n-hexanol increased in CS but decreased in PM, and propionic acid increased only in infected PM. On the other hand, in the grapes with anthracnose, heptanal and methanol decreased greatly in CS, and some substances, such as 1-penten-3-ol, n-hexanol, and propanal were raised in CS while decreased in PM. According to the results, it is speculated that the specific marker VOCs can be further found for specific varieties infected with specific pathogenic fungi. Finally, there was the same change rule in the two grapes, in which the content of hexanal and (*E*)-2-hexenal decreased and acetone increased for all infected grapes.

#### Effects of Pathogenic Fungal Infestations on Various Types of Volatile Organic Compounds

Heatmap with clustering analysis was shown to clarify the changes of all VOC among the diseases-affected grapes and the control group. In Figure 3C, each row showed volatile substances, and M, D, and T in parentheses behind the name of the substance represent the monomer, dimer, and trimer of the substance, respectively. Each column indicated different samples, and the number indicated temporarily unknown compounds. We compared the color difference to determine the variation of VOCs. Blue represents low concentration and red represents high concentration; the more obvious the color was, the more VOC was in the corresponding sample. The samples clustered into the same category indicated a high degree of correlation.

##### The Changes in C6-Volatile Organic Compounds

In VOCs, green leaf aroma C6-VOCs as one of the main families are derived from the lipoxygenase (LOX) pathway, which is usually induced by biological stress, the precursors are linoleic acid and α-linolenic acid (Gong et al., 2019). In this study, hexanal, (*E*)-2-hexenal, and 1-hexanol are typical C6-VOCs. Among them, hexanal and (*E*)–2-hexenal are C6 unsaturated aldehydes and 1-hexanol is C6 alcohol, which have the characteristics of green grass flavor. The concentrations of hexanal and (*E*)-2-hexenal in all infected grapes decreased, and C6-VOCs were generally associated with plant defense behavior. Shiojiri et al. (2006) showed that overexpression of hydroperoxide lyase (HPL) in Arabidopsis led to higher resistance of transgenic plants to *Botrytis cinerea*, which may be due to the increasing contents of C6 VOCs emitted by plants after infection, reflecting the assumed role of VOCs metabolism in grape defense mechanism. However, our results are in agreement with those reported by Santos et al. (2022), who found that hexanal and (*E*)-2-hexenal decreased greatly both in free or glycosylated after infection with *Botrytis cinerea*, suggesting that C6-VOCs can be used as a stress signal for plant biological stress. Schueuermann et al. (2018) also confirmed the concentration of (*E*)-2-hexenal in *V. vinifera* cv. Chardonnay grapes decreased after infection with *Botrytis cinerea* in two out of three vintages. We speculated that pathogenic fungal infestation could manipulate the level of C6-VOCs to reduce the defense effect of fruit, because low concentrations of (*E*)-2-hexenal may promote mycelium growth (Santos et al., 2022). Moreover, previous studies also showed that green aroma gradually decreased with fruit maturation, which was also a possibility for the decrease of hexanal and (*E*)-2-hexenal (Agudelo-Romero et al., 2013). Finally, these changes in C6-VOCs are likely to affect the green aroma of grapes.

##### The Changes in Alcohol

After pathogenic fungal infestations, alcohol-VOCs also changed greatly. Alcohols have physical-chemical properties, which can lead to membrane rupture and interfere with cell metabolism (Yalage Don et al., 2020). In this study, the results showed that 3-Methyl-1-butanol only increased in infected CS, 3-Methyl-1-butanol has the aroma of fusel alcohol and antibacterial properties. Pinar et al. (2017) showed that its presence is usually related to the activity of laccases in *B. cinerea*. Moreover, the existence of fusel alcohol is usually one of the reasons for the rotting smell of grapes. On the other hand, we also observed that increasing ethanol only in PM after pathogenic fungal infestation, and it was at a high level. we speculated that spontaneous fermentation could explain it because there were more yeasts in the microbial group of infected grapes compared with healthy grapes (Magyar, 2011).

##### The Changes in Ester Acetates

Esters play an important role in wine aromas, as most esters present pleasant aromas. The results showed that acetate accounted for a large proportion of VOCs in this study. Ethyl acetate showed higher levels only in CS after pathogenic fungal infestation, which always contains ethyl acetate. Ethyl acetate presents a strong fruit flavor, which is beneficial to the production of acetic acid, it usually acts as a common adverse metabolite that exists in fruit infected by fungi (Barata et al., 2011). We speculated that infected grapes produce a certain degree of fermentation, and the increase of acetate compounds may promote the colonization of fungi (Boss et al., 2015). In the PM infected with gray mold, white rot, and anthracnose, esters were decreased by 8, 7, and 6, respectively. Only 2 esters increased, which contains ethyl isobutyrate. Ethyl isobutyrate is formed by esterification of ethanol and isobutyrate under acidic conditions, which has an apple aroma. Ethyl isobutyrate may be a specific biomarker for PM after pathogenic fungal infestation, which needs further confirmation in the following experiment.

##### The Changes in Aldehydes and Acids

In CS, heptanal decreased remarkably and acetic acid increased after pathogenic fungal infestation. Usually, acetic acid is the symbolic volatile of fruit decomposition and decay, which is the pathway to produce ethyl acetate and may be caused by the co-infection of acetic acid bacteria and acid rot (Hall et al., 2018).

##### The Changes of Unknown Volatile Compounds

In this study, 13 volatile compounds were unidentified from the fingerprints due to information limitations in the built-in NIST database of GC-IMS. The unknown volatile compounds were analyzed deeply for the integrity and reliability of the experiment. Terpenes and norisoprenoids are two of the most important aromatic chemicals found in grapes, both in volatile and non-volatile forms, and are known for contributing fruity or flowery notes (González-Barreiro et al., 2015). However, these compounds are found in low concentrations as most of them have very low perception threshold levels (Diéguez et al., 2003). The results showed that the detected unknown compounds had low concentrations, so we speculated that these compounds might be terpenes and norisoprenoids. In CS and PM after infection, compound 10 increased, compound 6 decreased, and compounds 4, 5, 7, and 12 did not change greatly; other numbered compounds may not change consistently and may be due to varietal differences. Among them, in grapes suffering from a pathogenic fungal infestation, compound 11 increased in PM and slightly decreased in CS and compounds 1, 2, and 3 decreased in CS but did not change more in PM.

#### Differences in Volatile Organic Compounds Present in Different Varieties of Grapes Resulting From Pathogenic Fungal Infestations

**FIGURE 4:**
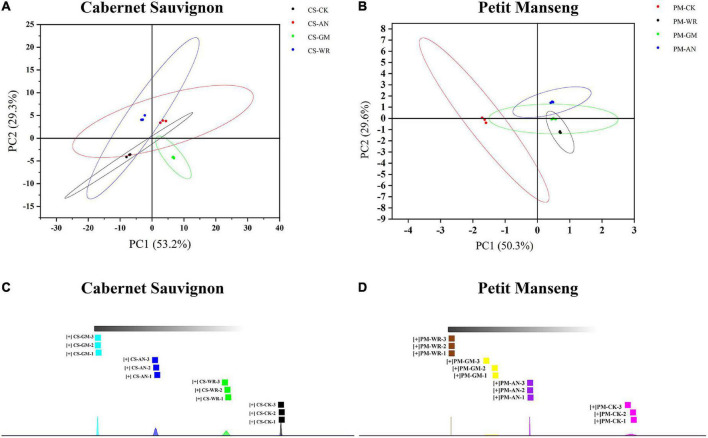
**(A, B)** Principal component analysis (PCA) score plot and **(C, D)** nearest neighbor fingerprint (NNA) of Cabernet Sauvignon and Petit Manseng wine grapes. The bottom area of NNA shows the normal distribution of each sample.

The principal component analysis (PCA) and nearest neighbor fingerprint (NNA) (Figures 4A–D) intuitively showed the differences between different samples. Different color points represent different samples, the greater the distance between sample points is, the greater the difference is. The PCA of volatile compounds in both healthy and different infected samples was demonstrated in Figures 4A,B. The accumulative contribution of the first and second principal components in CS and PM was 82.5% (PC1 was 53.2% and PC2 was 29.3%) and 79.9% (PC1 was 50.3% and PC2 was 29.6%), respectively. The score map clearly illustrated the PCA, comparing the healthy and infected grapes of CS and PM; where the PC1 score variation could be considered as the positive and negative ranges. Meanwhile, the difference in infected samples with different pathogenic fungi could be separated by the different scores of PC1 and PC2. Moreover, the NNA was conducted for further analysis. The fingerprint of the aromatic components of CS and PM was shown in Figures 4C,D, according to the similarity of aroma profiles, the samples were divided into various groups. The results showed that the VOCs of CS-GM had the most significant difference compared with others. The difference between VOCs in CS was as follows: gray mold > anthracnose > white rot, while the difference between VOCs in PM was as follows: white rot > gray mold > anthracnose. Also, the VOCs of CS had more alterations after infection than PM. It is not difficult to explain because of the difference in varieties. The strong antibiosis resistance and freshness of PM may indicate that the VOCs have fewer changes after infection, and the total phenolics, total flavonoids, and tannins of healthy grapes in CS are higher than in PM. Therefore, it is normal to explain the different results when two grape varieties were infected with the same pathogenic fungal.

## Conclusion

In this work, the effect of different pathogenic fungal infestations was investigated on berry quality and VOCs of CS and PM. The quality changed after pathogenic fungal infestation, including the content of VC, TF, and tannins, showed a downward tendency in most of the infected grapes, which is likely because the infestations interfered with the normal physiological metabolism of grapes and changed their composition. Meanwhile, higher levels of TA only appeared in disease-affected CS, and SSC decreased in disease-affected CS but increased in a disease-affected PM; these inconsistent results were speculated to be due to various factors such as varieties and resistance. The results of 100-berry weight showed that there was no significant difference, indicating that pathogenic fungal infestations had little effect on it. The VOCs were investigated by GC-IMS to determine the types and comparative content, a total of 61 VOCs were identified and then investigated from different functional groups, including C6-VOCs, alcohols, ester acetates, aldehydes, and acids. Hexanal and (*E*)-2-hexenal decreased in all infected grapes, which may be due to the C6-VOCs being manipulated to reduce the defense effect of the berry after pathogenic fungal infestation. In disease-affected CS, a higher level of 3-Methyl-1-butanol was related to the activity of laccases in *B. cinerea.*, and only increasing ethanol in disease-affected PM, which was speculated that spontaneous fermentation could explain it because there were more yeasts after infection. These unique VOCs may serve as hypothetical biomarkers to help us identify specific varieties of pathogenic fungal infestations. Furthermore, the results of PCA and NAA showed differences in VOCs present in different varieties of grapes resulting from pathogenic fungal infestation, which indicated that the VOCs of CS changed more than PM after infection, and the VOCs produced by different pathogenic fungal infestations were also different. The difference between VOCs in CS was as follows: gray mold > anthracnose > white rot, while the difference between VOCs in PM was as follows: white rot > gray mold > anthracnose. Finally, this study is beneficial for us to strengthen the understanding of pathogenic fungal infestations during the growth and development of grapes and explore the interaction between pathogenic fungal infestations and grapes. However, the mechanism of VOCs between grapes and pathogenic fungal infestations still needs further research.

## Data Availability Statement

The raw data supporting the conclusions of this article will be made available by the authors, without undue reservation.

## Author Contributions

XYL: methodology, data curation, and writing the original draft preparation. TL: data curation, resources, and formal analysis. ML: conceptualization, software, and writing the original draft preparation. DC: visualization and investigation. XWL: software and resources. SZ: formal analysis and data curation. XD: conceptualization and validation. JC: supervision, formal analysis, and resources. ZK: conceptualization, resources, writing, reviewing, and editing, project administration, and funding acquisition. JT: validation, writing, reviewing, and editing, supervision, and project administration. All authors contributed to the article and approved the submitted version.

## Conflict of Interest

The authors declare that the research was conducted in the absence of any commercial or financial relationships that could be construed as a potential conflict of interest.

## Publisher’s Note

All claims expressed in this article are solely those of the authors and do not necessarily represent those of their affiliated organizations, or those of the publisher, the editors and the reviewers. Any product that may be evaluated in this article, or claim that may be made by its manufacturer, is not guaranteed or endorsed by the publisher.
